# Detection of Laurel Wilt Disease in Avocado Using Low Altitude Aerial Imaging

**DOI:** 10.1371/journal.pone.0124642

**Published:** 2015-04-30

**Authors:** Ana I. de Castro, Reza Ehsani, Randy C. Ploetz, Jonathan H. Crane, Sherrie Buchanon

**Affiliations:** 1 Citrus Research and Education Center, University of Florida/IFAS, 700 Experiment Station Road, Lake Alfred, FL 33850, United States of America; 2 Tropical Research and Education Center, University of Florida/IFAS, 18905 SW 280th St, Homestead, 33031 Florida, United States of America; Gyeongnam National University of Science and Technology, KOREA, REPUBLIC OF

## Abstract

Laurel wilt is a lethal disease of plants in the Lauraceae plant family, including avocado (*Persea americana*). This devastating disease has spread rapidly along the southeastern seaboard of the United States and has begun to affect commercial avocado production in Florida. The main objective of this study was to evaluate the potential to discriminate laurel wilt-affected avocado trees using aerial images taken with a modified camera during helicopter surveys at low-altitude in the commercial avocado production area. The ability to distinguish laurel wilt-affected trees from other factors that produce similar external symptoms was also studied. R_mod_GB digital values of healthy trees and laurel wilt-affected trees, as well as fruit stress and vines covering trees were used to calculate several vegetation indices (VIs), band ratios, and VI combinations. These indices were subjected to analysis of variance (ANOVA) and an M-statistic was performed in order to quantify the separability of those classes. Significant differences in spectral values among laurel wilt affected and healthy trees were observed in all vegetation indices calculated, although the best results were achieved with Excess Red (ExR), (Red–Green) and Combination 1 (COMB1) in all locations. B/G showed a very good potential for separate the other factors with symptoms similar to laurel wilt-affected trees, such as fruit stress and vines covering trees, from laurel wilt-affected trees. These consistent results prove the usefulness of using a modified camera (R_mod_GB) to discriminate laurel wilt-affected avocado trees from healthy trees, as well as from other factors that cause the same symptoms and suggest performing the classification in further research. According to our results, ExR and B/G should be utilized to develop an algorithm or decision rules to classify aerial images, since they showed the highest capacity to discriminate laurel wilt-affected trees. This methodology may allow the rapid detection of laurel wilt-affected trees using low altitude aerial images and be a valuable tool in mitigating this important threat to Florida avocado production.

## Introduction

Laurel wilt is a lethal disease of some native and non-native species in the Lauraceae family in the southeastern United States, including an important agricultural commodity, avocado (*Persea americana*) [1, 2]. *It is caused by the Asian fungus* Raffaelea lauricola *and* has ambrosia beetle vectors, including *Xyleborus glabratus*. *Xyleborus g*. was first reported in the Western Hemisphere in 2002 in Port Wentworth, GA [3, 4]. Laurel wilt has since spread rapidly along the southeastern seaboard of the United States. In February 2011, laurel wilt was confirmed for the first time in Miami-Dade County, 10 miles north of Florida's main avocado production area in Homestead [5]. In 2012, it was first detected in the commercial avocado production area (CAPA) [6]. The rapid spread, in less than a decade along much of the coastal plains of the southeastern United States and as far west as southern Mississippi [7], could be explained by the prevalence of highly susceptible native hosts, such as redbay (*P*. *borbonia*) and swampbay (*P*. *palustris*), natural dissemination of *X*. *glabratus*, and anthropogenic movement of vector-infested materials [8].

Laurel wilt is a vascular disease that plugs the xylem, thereby impeding the flow of water and nutrients in affected trees. External symptoms of wilting and foliar necrosis develop rapidly in affected portions of the tree, and avocado trees defoliate within 2–3 months of symptom onset [8]. Many of these symptoms are similar to those that are caused by other diseases or factors, such as freeze damage, Phytophthora root rot, Verticillium wilt, lightening and fruit stress (overbearing) and thus, can be difficult to distinguish visually [9]. Moreover, since external symptoms develop only after significant colonization of the host [8], it may not be possible to manage the disease once plants display external symptoms [10]. Thus, the ability to detect laurel wilt before the onset of external symptoms would be invaluable when managing this disease [11].

Approximately 4.4 million tons of avocado were harvested worldwide in 2012 [12]. The projected harvest in 2014 amounts to 3.9 million tons; and the U.S. and France are expected to be the largest importers (41% and 27% of the total, respectively). According to the NASS (2012) [13], California accounts for the majority of U.S. avocado production, followed by Florida and Hawaii. While commercial production in California occurs in in four counties, in Florida, it is highly localized, with 98% grown in south-eastern Miami-Dade County [8].

Other diseases can kill avocado trees, but none of them develop as quickly as laurel wilt [8]. This devastating disease has the potential to destroy avocado production in Florida, and in the absence of a reliable control strategy, could cause losses of $27 to 54 million [1, 8]. Since laurel wilt is a recent disease, continuous research on disease detection, fungicide application, vector control, sanitation (elimination of affected trees) and development of tolerant varieties is necessary [14].

Sanitation, in which laurel wilt-affected avocado trees are identified and destroyed before new generations of vectors emerge and colonize new host trees, has become an important tool for battling laurel wilt. Detection is a first and important step in effective sanitation, but accurate and rapid measures are lacking [9]. The current diagnostic method involves visual inspection of suspect trees, collection of symptomatic wood, and lab analyses. A rapid technique to replace this time-consuming and expensive method could be quite useful in mitigating the development and spread of this disease [11].

Remote sensing tools can significantly improve disease detection only if the spectral and spatial resolution of remote sensing equipment is sufficient for the detection of differences in spectral reflectance [15]. Spectroscopy in the visible and near infrared ranges has been found to be a suitable technique for disease detection, including laurel wilt [16, 9]. Other approaches have included the use of multispectral imagery to detect, monitor, and quantify diseases of tomato [17], winter wheat [18], creeping bentgrass (*Agrostis stolonifera* var. palustris Farwell) [19], cranberries [20], olives [21], and citrus [22–24]. Usha and Singh (2013) [25], Sankaran *et al*. (2010) [11], and Barton (2012) [26] reviewed the potential for image-based remote sensing to detect crop diseases. García-Ruiz *et al*. (2013) [23] compared a multi-band imaging sensor attached to a UAV with a similar hyperspectral imaging system (aircraft-based sensors) to detect Huanglongbing (HLB) in citrus. They achieved better accuracies in classification results when multi-band images where used (67–85%), demonstrating the potential for high-resolution aerial detection of HLB. Calderón *et al*. (2013) [21] explored the use of multispectral imagery to detect damage caused by Verticillium wilt in olive and reported that some visible ratios detected early stages of disease development. However, to the best of our knowledge, multi-spectral aerial imagery has not been used in laurel wilt.

As part of an overall research program to suppress laurel wilt in Florida’s CAPA, spectral criteria have been sought for the quick, economic, and accurate detection of the disease. In a previous study, visible-near infrared spectra were used to discriminate symptomatic and healthy leaves, proving the potential for visible-near infrared detection of laurel wilt [9]. The main objective of the present study was to evaluate the possibility of discriminating laurel wilt-affected avocado trees using multi-band aerial images taken at low altitude. The specific goals were: a) select the best vegetation indices to discriminate efficiently between healthy and laurel wilt-affected trees, b) evaluate the feasibility of separating laurel wilt from other factors that produce external symptoms similar to laurel wilt.

## Material and Methods

### Study area and data acquisition

Periodic helicopter surveys for laurel wilt were conducted over 150 square miles in the CAPA in south Miami-Dade County to demarcate the presence and spread of laurel wilt in this area. During these surveys, modified color aerial images (R_mod_GB: blue, B: 390–520 nm; green, G: 470–570 nm; red-edge, R_mod_: 670–750 nm) were taken with a point-and-shoot camera (a modified Canon SX260 NDVI, Canon U.S.A., Inc. Melville, NY, USA). The Canon SX260 NDVI camera was modified to capture information in both red-edge and visible light (green and blue), through adding a 37-mm filter ring to the front nose of the camera, manufactured by the company LDP-LLC (LDP-LLC, Carlstadt, NJ 07072 USA), where a calibration process was carried out. This model is capable of acquiring 12.1 megapixel spatial resolution images with 8-bit radiometric resolution and is equipped with a 5.7–18.8 mm zoom lens. The images were saved in JPG format and stored on a secure digital (SD) card.

**Fig 1:**
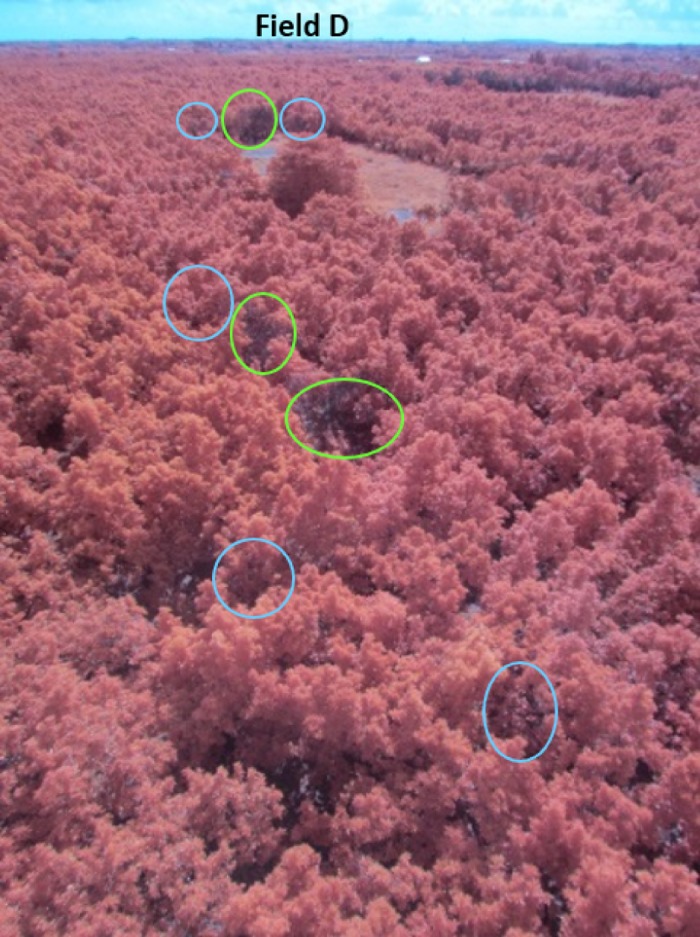
False color aerial image captured in Field D. The circles in green color represent avocado trees showing advantage stage symptoms of laurel wilt disease. The circles in blue color represent avocado trees with initial symptoms of laurel wilt affection.

**Fig 2:**
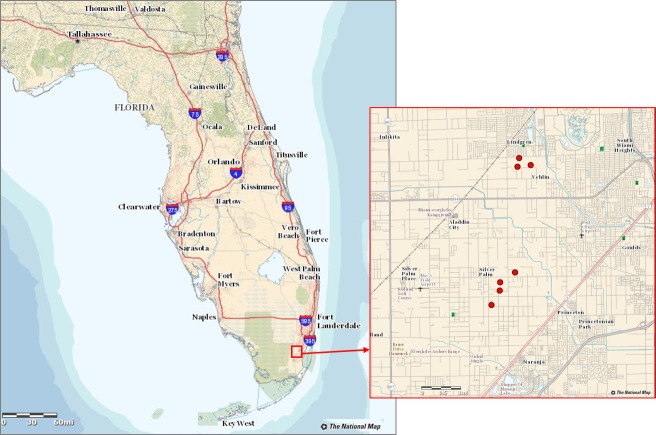
Location of the study area in Florida State and Miami Dade County.

**Fig 3:**
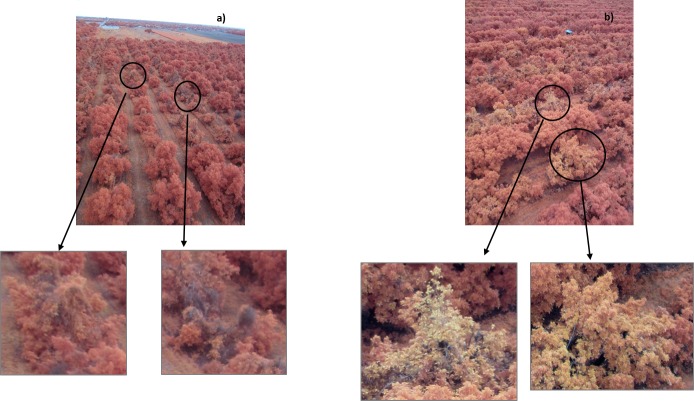
False color images of a) vines and b) fruit stress trees. The circles show details of represented factors and they are shown below in a zoom.

During surveys conducted in October 2013, 65 aerial images were taken in six locations with an average flight height of 60 m above the ground (Fig 1). These locations were identified as fields A (latitude and longitude coordinates, 25.552879, -80.427540), B (25.548916, -80.430335), C (25.593048, -80.424993), D (25.593172, -80.422209), E (25.595593, -80.424547), F (25.549920, -80.431738) and G (25.540705, -80.435734) (Fig 2). The flights were authorized by a written agreement between the farm owners and our research group. Suspect trees identified during the field surveys were diagnosed and compared with those affected by other diseases and factors that cause similar symptoms; trees struck by lightning, damaged by frost, or affected by phytophthora root rot look very similar to those affected by laurel wilt. All putative isolates of *Raffaelea lauricola* were identified based on their colony phenotype on CSMA+ [8], and a representative subset of isolates were confirmed as the pathogen with SSR molecular markers [27]. Regions of interest of healthy trees and those affected by laurel wilt were selected from the images corresponding to certain fields (A, C, D, E, F and G), and digital values of both classes were extracted. Data from other factors that cause similar symptoms, as seen from the helicopter, such as fruit stress and vines covering avocado, were found in some of those fields and were extracted from images (Fig 3). Pixels of the images presented digital counts within the range of 0–255. ENVI software (ENVI, Research Systems Inc., Boulder, CO, USA) was used to process and analyze the images.

### Data analysis

R_mod_GB spectral values of healthy avocado trees and those affected by laurel wilt were used to calculate several vegetation indices (VIs), band ratios (B/G; R/G; (R—G)), and VI combinations from them. Only information drawn from the images was used, without any preprocessing. In similar studies, good results were achieved when simple raw pixel values were used and matched to VIs calculated from ground truth samples collected in the field [28, 29].

The following VIs were used in this study:

- *R/B index* [30]
  $$R/B=\frac{R_{mod}}{B}\;$$ (1)
- Green vegetation index (VI green, [31])
  $$VIgreen=\frac{G-R_{mod}}{G+R_{mod}}\;$$ (2)
- *Excess blue* [32]
  ExB=1.4B−G  (3)
- *Excess green* [33, 34]
  $$ExG=2G-R_{mod}-B$$ (4)
- *Excess red* [35]
  $$ExR=1.4R_{mod}-G\;$$ (5)
- *Normalized pigment chrolophyll index* [36]
  $$NPCI=\frac{R_{mod}-B}{R_{mod}+B}\;$$ (6)
- *Color index of vegetation* [37]
  $$CIVE=0.441R_{mod}-0.811G+0.385B+18.78745$$ (7)
- *Vegetative* [38]
  $$VEG=\frac{G}{R_{mod}^{a}B^{\left(1-a\right)}}\;\;\;\;\text{with a set to 0.667 as in its reference}$$ (8)
- *Woebbecke index* [33]
  $$WI=\frac{G-B}{R_{mod}-G}$$ (9)
- *Excess green minus excess red* [39]
  $$ExGR=ExG-1.4R_{mod}-G$$ (10)

VI Combinations used:

- *Combination 1* [32]
  COMB 1=0.25ExG+0.3ExGR+0.33CIVE+0.12 VEG (11)
- *Combination 2* [40]
  COMB 2=0.36ExG+0.47CIVE+0.17 VEG (12)

A correlation analysis was carried out for all proposed vegetation indices using Hoeffding’s D measure [41] to investigate the statistical strength of relationship between each pair of variables and identify the mutual dependence of those vegetation indices. The statistic ranges between -0.5 and 1.0; the higher the value of D, the more dependent the variables are on each other. The calculation of Hoeffding's D measure was performed with the JMP 10 (SAS Institute Inc., Campus Drive, Cary, NC, USA 27513).

The above independent VIs corresponding to healthy and laurel wilt trees were subjected to analysis of variance (ANOVA) at the 0.01 level of significance by a Tukey Honestly Significant Difference (HSD) range test. JMP software was employed to perform the statistical analysis. The same vegetation indices and ANOVA technique were used with laurel wilt and other factors and disease data found in those images.

The M-statistic (Eq 13), originally presented by Kaufman and Remer (1994) [42] was performed in order to quantify the separability of healthy and laurel wilt trees depending on VIs. M expresses the difference in the means of two classes of histograms normalized by the sum of their standard deviations (σ). The same difference in means will give different measures of separability depending on the spread of the histograms; wider histograms (larger σ) will cause more overlap and less separability than narrow histograms (smaller σ) for the same difference in means [42]. The authors reported that a value of M < 1.0 indicates poor separation, while M > 1.0 indicates good separation, showing easier separation for larger M values [43].

$$\;\;M=\frac{Mean\;Diff}{Sum\;Std\;deviation}\;=\;\frac{\mu_{a\;-}\mu_{b}}{\sigma_{a}+\sigma_{b}}$$ (13)

R_mod_GB spectral values of fruit stress trees and vines covering healthy trees were extracted from images and compared with a healthy and laurel wilt-affected set of data obtained from a 10% randomly selected RGB data for every field. The best vegetation indices in the previous step were used to perform ANOVA at the 0.01 level of significance by a Tukey HSD range test, as well as the M-statistic. A box plot was drawn to show significant spectral differences among healthy, laurel wilt-affected, fruit stress, and vines covering trees.

## Results and Discussion

### Laurel wilt-affected and healthy trees discrimination

Significant differences in spectral data between laurel wilt-affected and healthy trees were observed in all VIs calculated with the exception of the Vegetative Index in field A (data not shown). These results confirm the potential of discriminating laurel wilt-affected trees from healthy trees in the RGB region of the spectrum using aerial images taken at a low-altitude from a helicopter. Avocado healthy plants show a green color while laurel wilt-affected leaves turn brown after wilting. The vegetation indices used in this work take advantage of differences in the reflectance of vegetation between wavelengths, accentuating a particular color, which is of interest [44].

The best results obtained with the M-statistic were ranked and are shown in Table 1. M values varied according to the location and vegetation indices, achieving values > 1.0 in all locations when ExR, (R—G), R/B, B/G, and COMB1 were used. M values higher than 1.0 indicate good separation, suggesting satisfactory results for vegetation discrimination [45]. M values for vegetation indices not shown in Table 1 achieved values lower than 1 in all locations, showing more a difficult degree of spectral separation between healthy and laurel wilt trees. Thus, although significant spectral differences were achieved with those VIs, a poor spectral separation is expected between healthy and laurel wilt-affected avocado trees.

**Table 1:** M-statistic obtained for the best VI in each field.

	Field A	Field C	Field D	Field E	Field F	Field G
ExR	**1.6**	**2.8**	**4.4**	**3.1**	**2.6**	**3.5**
R—G	**1.4**	**2.6**	**3.6**	**2.3**	**1.9**	**3.0**
COMB1	**1.2**	**2.2**	**3.2**	**2.0**	**1.6**	**2.6**
R/B	**1.3**	**1.9**	**2.5**	**1.9**	**1.7**	**2.0**
B/G	1.0	1.2	1.6	1.4	1.5	1.3
WI	1.2	1.6	2.1	0.7	0.80	0.9
COMB2	0.8	1.5	2.0	1.1	0.6	1.6

The values given in bold represent the vegetation indices that achieve values >1.3 in all locations, except COMB 1 in Field A.

Other vegetation indices, such as WI and COMB2 acquired M-statistic values higher than 1 in some fields, e.g. M values of 2.1 in field D and 1.6 in field C using WI; however, they showed a poor capacity of separation in the other fields, with M values lower than 1, e.g. 0.8 and 0.6 using COMB2 in field A and F, respectively. Therefore, those vegetation indices do not show a robust behavior in all scenarios.

Smith *et al*. (2007) [43] selected only those indices that achieved an M-statistic value greater than an arbitrary value to enable a more targeted analysis subset. Overall larger values of M (> 1.3) were found using ExR, (R-G), COMB1 and R/B in all locations. These consistent results confer a high robustness of healthy and laurel wilt-affected trees discrimination using those VIs in all of the avocado field locations sampled. ExR performs significantly better than all other indices in all locations, with the next best discriminator being the (R-G), followed by the COMB1 and R/B.

ExR was the index that showed the best spectral separability in all fields, reaching the highest M values, e.g. 3.06 in field F and 4.34 in field D. ExR is a redness index, which emphasizes the color of laurel wilt-affected leaves, allowing the best discrimination between them and green healthy leaves. Guijarro et al. 2011 [32] used this index to identify soil in RGB images. Regarding these consistent results, the ExR index should be the first index used in the classification of LW-affected and healthy trees in further research.

The R-G index was found to be the second best index with higher values in all studied locations. These results may be explained by taking into account that this disease plugs the xylem, impeding the flow of water and nutrients in affected trees, resulting in an increase in tree temperature. For this reason, the amount of chlorophyll in the leaves is reduced and cell structure is damaged, so the reflectance in the red-edge and near infrared (from 700 to 1100 nm) regions decreases considerably. Thereby, the absolute difference between red-edge band (670–750 nm) and the green band (470–570 nm) decreased in laurel wilt-affected plant spectral data. Green and red bands were studied by Gitelson et al. (2002) [31] in order to quantify the relations between them, the vegetation fraction and chlorophyll absorption.

Combination 1 (COMB 1) was introduced by Guijarro *et al*. (2011) [32] to determinate greenness in images, which combines the information provided by four green indices ExG, ExGR, CIVE, and VEG. The weight associated with each green index was the one that obtained the lowest percentage of error in discrimination. Therefore, we achieved high M-values in discrimination between healthy-green plants and laurel affected plants using COMB 1 in every field analyzed, since this combination allows increasing contrast between green plants and other land uses, improving discrimination. According to Guijarro *et al*. (2011) [32], COMB 1 outperformed the simple green vegetation index in all locations.

The fourth best M values were achieved in most of the locations with R/B. This index uses the blue and red-edge band information. These results were in agreement with those of Peña-Barragán *et al*. (2007) [46] and de Castro *et al*. (2012) [47], who reached accurate classification results with R/B index when the ground covers analyzed turned different colors, and this index enhanced those differences.

Contrast between red-edge, green, and blue reflectance was used to analyze the discrimination between healthy and laurel wilt affected avocado trees. Gitelson *et al*. (2002) [31] found that for vegetation fractions of more than 60%, the information content of reflectance spectra in the visible range can be expressed by only two independent pairs of spectral bands: the blue and the red, and the green and the red-edge region. In the same vegetation fraction, the RGB vegetation indices used by Gitelson *et al*. (2002) [31] showed higher sensitive than for Normalized Difference Vegetation Index.

Therefore, our findings indicate that is possible discriminate laurel wilt-affected trees using R_mod_GB aerial images taken at low altitude with high capacity, with ExR being the vegetation index that best performs this discrimination.

### Other factors and laurel wilt-affected trees discrimination

**Fig 4:**
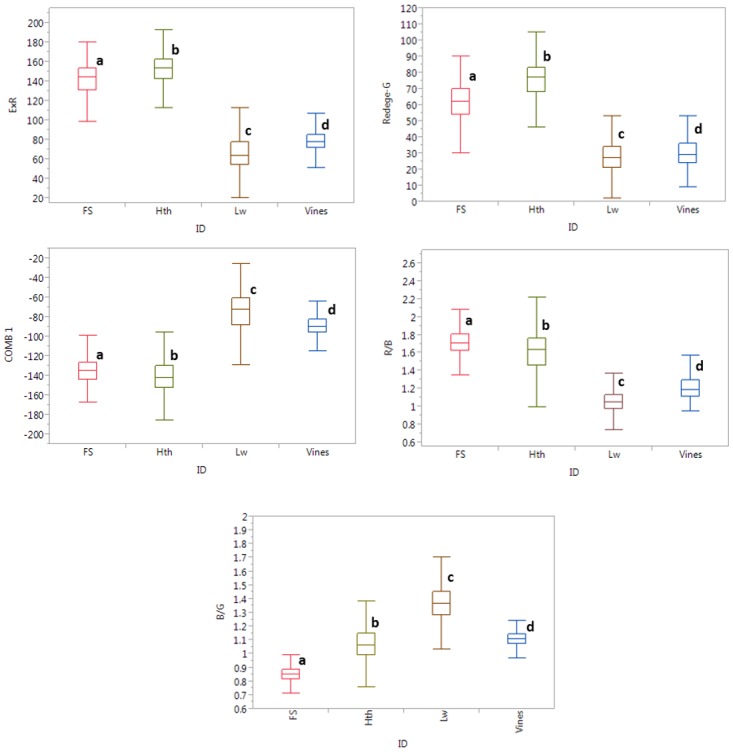
Box plot showing variation in vegetation index selected for healthy and laurel wilt affected plants. Box plots followed by different letter are significantly different according to Tukey HSD test at a 0.01 level of significance.

The box plot in Fig 4 shows a comparison of the ability of ExR, (R-G), COMB1, R/B, and B/G to separate healthy, laurel wilt-affected trees, fruit stress, and vines covering trees. All of the vegetation indices were significantly different for each studied class, so any of the indices could make the separation. Spectral differences were higher between healthy plants and laurel wilt-affected trees in all vegetation indices, so those vegetation indices provide enough robustness for detecting laurel wilt-affected trees since a combination of data from all locations was used. However, the overlapping of values in some box plots between laurel wilt-affected trees and vines and fruit stress and healthy plants could make the separation between them more difficult. Although a fruit stressed avocado tree looks like an affected laurel wilt tree because of wilting leaves, it is actually a healthy tree, so it shows similar spectral information to that of the healthy class. On the other hand, a vine covered tree also looks like a laurel wilt-affected tree, but in this case, the color of those plants are browner, so it has spectral information closer to that of a laurel wilt-affected tree. From an agronomic point-of-view, the separation between healthy and fruit stress plants is not a problem, since the objective was to discriminate laurel wilt in avocado orchards, and great spectral differences were found between fruit stress plants and laurel wilt-affected trees in all of the vegetation indices.

The M-statistic was perfomed to check if the spectral difference between laurel wilt-affected trees and vines were enough to separate these classes. M-values achieved values > 1.3 only when B/G was used (1.53), while values lower than 1.0 were obtained using the rest of the vegetation indices analyzed (Table 2). Vines have a more similar reflectance between R_mod_, G and B bands than green vegetation, since reflectance of G and R_mod_ bands in vines are more smoothed compared to green vegetation. Those fewer spectral differences between R_mod_GB bands make vines a natural brownish color, while a higher spectral differences between R_mod_GB allow green vegetation show this color. On the other hand, laurel wilt-affected trees showed blue and green values that were more similar to healthy plants. Thereby, the B/G index displayed a very good capacity to separate laurel wilt-affected trees from vines, allowing discrimination between both classes and becoming a required VI to perform in further classification.

**Table 2:** M-statistics obtained comparing laurel wilt and vines.

	M values
**B/G**	**1.53**
R/B	0.57
COMB 1	0.47
ExR	0.43
R—G	0.10

The value given in bold represent the vegetation indices that achieve value >1.3

These consistent results prove the usefulness of the R_mod_GB camera in discriminating laurel wilt-affected avocado trees from healthy plants, as well as from other factors that cause the same symptoms as this disease and suggest performing the classification in further research. According to our results, ExR and B/G showed the highest capacity, so they could be used to develop a specific algorithm or decision rules to classify aerial images. This inexpensive and easy-to-handle camera may allow the rapid diagnosis of laurel wilt-affected trees using low altitude multi-band aerial images by classifying large avocado field areas. In addition, a higher spectral resolution camera, i.e. higher number of bands and narrower wavelengths, should be tested in order to reduce the overlapping of values in the boxplot between laurel wilt-affected trees and vines, if classification results are not highly satisfactory.

Early information of laurel wilt disease will facilitate the control of this disease through proper management strategies, since its rapid development makes it very difficult to manage [10]. Thereby, farmers may control the movement of this devastating disease and keep the Florida CAPA in the second position of avocado production in US, as well as achieve the value predicted by the FAO, where the US will be a major producer of avocado.

## Conclusions

As part of an overall research program to detect and suppress laurel wilt in Florida’s CAPA, the possibility of discriminating laurel wilt-affected trees using RGB aerial images taken at low altitude was evaluated. The analysis of these images showed that different VIs can be used to detect trees affected by laurel wilt.

The best results were achieved with ExR, (R-G), COMB1, and R/B indices in all studied locations. These consistent results showed a robust discrimination between healthy and laurel wilt-affected trees, as well as the usefulness of modified camera (R_mod_GB) in further research. The ExR index performed significantly better than all other indices in all locations; (R-G) was the next best discriminator, followed by the COMB1 and R/B indices.

Analysis of fruit stressed avocado trees and vines, combined with a random sample of laurel wilt-affected trees and healthy plants from all locations analyzed, showed significant differences when best vegetation indices obtained in the previous discrimination were used. However, only B/G displayed a high capacity of separation between laurel wilt-affected trees and other factors, becoming a required vegetation index to perform in further classification.

The results found in this research confirm the potential to discriminate laurel wilt-affected trees from healthy plants and other factors that cause same symptoms, such fruit stress and vines, using aerial R_mod_GB images taken at a low-altitude from a helicopter. The contrast between red-edge, green, and blue reflectance were found to be enough for the highly accurate discrimination of laurel wilt-affected avocado from those classes. According to our results, ExR and B/G should be utilized to develop an algorithm or decision rules to classify aerial images, considering that both indices showed the highest capacity to discriminate laurel wilt-infested trees.

This inexpensive and easy-to-handle camera will enable a rapid and accurate assessment of laurel wilt disease development and progress in study areas, as well as provide a valuable tool in mitigating this important threat to Florida avocado production. The authors suggest trying a higher spectral resolution camera, i.e. one with a higher number of bands and narrower wavelengths, to test if better results can be achieved in further classification research.

The use of early information about laurel wilt disease will help farmers to control the movement of this disease through proper management strategies. Development of the methodology proposed in this research would allow farmers to maintain avocado production as one of most important crops in Florida, in addition to achieving that value predicted by the FAO where USA will be will be a major producer of avocado in 2015.
